# Risk and resilience: adverse and positive childhood experiences and aggression in adults with and without ADHD

**DOI:** 10.3389/fpsyt.2026.1759667

**Published:** 2026-03-10

**Authors:** Johannes Merscher, Wolfgang Retz, Daniel Turner, Petra Retz-Junginger, Steffen Barra

**Affiliations:** 1 Institute for Forensic Psychology and Psychiatry, Saarland University, Homburg, Germany; 2 Department of Psychiatry and Psychotherapy, University Medical Center of the Johannes Gutenberg University, Mainz, Germany

**Keywords:** ADHD, aggression, childhood maltreatment, protective childhood experiences, resiliency theory

## Abstract

**Background:**

Identifying risk and protective factors for aggressive behavior is central to effective violence prevention and public safety. In forensic psychiatry, attention-deficit/hyperactivity disorder (ADHD) is common among offenders and is linked to adverse childhood experiences (ACEs) on the one hand and increased aggression on the other hand. Yet, the mechanisms connecting these factors remain insufficiently understood. Evidence on the protective potential of positive childhood experiences (PCEs), particularly when considered alongside ACEs, is also limited.

**Methods:**

Guided by resilience theory and a compensatory resilience model, this study analyzed the dynamics among self-reported ACEs, PCEs, and current aggression in an ADHD subsample (n = 154) and a non-ADHD population (n = 205) using hierarchical linear regression analyses.

**Results:**

Compared with the non-ADHD group, adults with ADHD reported higher ACE loads, lower PCE scores, and greater aggressive tendencies. In both subsamples, ACEs significantly predicted higher aggression. Among individuals without ADHD, PCEs demonstrated an independent protective association with aggression after adjusting for ACEs and attenuated the ACE–aggression association. This compensatory effect of PCEs was not observed in the ADHD group. Overall, the harmful influence of ACEs on adult aggression appeared to outweigh any mitigating role of PCEs, particularly among individuals with ADHD.

**Conclusion:**

ACEs emerged as a robust correlate of current aggression in adults with and without ADHD, underscoring the need to systematically integrate developmental adversity into forensic risk assessment. For individuals with ADHD, violence prevention and public safety strategies may particularly benefit from early prevention and reduction of childhood adversity, trauma-focused interventions where indicated, and evidence-based ADHD treatment to limit the impact of ADHD-related impairments on dynamic aggression-related risk factors. For individuals without ADHD, prevention and rehabilitation efforts may be strengthened by simultaneously reducing ACEs and actively promoting PCEs as resilience-enhancing conditions.

## 1 Introduction

Over recent decades, adverse childhood experiences (ACEs), such as physical, emotional, or sexual abuse, neglect, household dysfunction, and adverse peer interactions, have emerged as a key construct for understanding how early adversity shapes health and development across the life course (1). Experiencing multiple ACEs is a significant risk factor for various health conditions, with the strongest associations linked to harmful alcohol use, illicit drug use, and interpersonal violence (2). Cross-sectional and longitudinal research consistently demonstrates a link between ACE exposure and various indicators of aggression and delinquency (3–10). A robust body of studies underscores a dose-response relationship between ACEs and crime risk, with each additional ACE significantly heightening the probability of serious, violent, and chronic offending (11, 12).

Individuals with attention-deficit/hyperactivity disorder (ADHD) frequently report elevated rates of ACEs as well as aggressive behavioral tendencies (13, 14). With prevalence rates of around 5.5% (15), ADHD, characterized by symptoms of inattention, hyperactivity and/or impulsivity (16), is among the most prevalent behavioral disorders in childhood and adolescence (17–20). In adulthood, prevalence estimates range between 2% and 3% (21, 22), with 2.6% of adults meeting criteria for persistent ADHD from childhood and 6.8% exhibiting clinically significant current ADHD symptoms (23).

ADHD is associated with substantial long-term adverse outcomes, including reduced educational success and academic achievement, interpersonal and relationship problems (24), an elevated risk of psychiatric comorbidities, and an increased likelihood of involvement in criminal behavior (21, 25–27). To explain the robust association between ADHD-related risk for persistent criminal behavior and aggressive outcomes, including interpersonal and intimate partner violence (13, 27–30), researchers have proposed several disorder-specific mechanisms. ADHD is conceptualized as a self-regulation disorder that includes difficulties in emotion regulation (31–34), which have been linked to increased aggressive behavior (35). Moreover, common deficits in executive functions, particularly inhibitory control, promote impulsive behavior and thereby contribute to aggressive and externalizing tendencies (36–38). Inhibitory control enables the regulation of attention, behavior, and cognitive processes allowing individuals to resist impulses and distractions in favor of goal-directed action. Deficits in inhibitory control increase reliance on habitual responses and sensitivity to environmental cues (39).

ACEs can induce toxic stress that impairs neurodevelopment, leading to alterations in key brain regions such as the prefrontal cortex, amygdala and hippocampus, with children exposed to threat frequently exhibiting reduced volumes in these stress- and emotion-regulation-related structures in non-clinical and general population samples (40, 41). Prolonged exposure to stress activates neurobiochemical feedback mechanisms that trigger the release of stress hormones (12, 42–45). Chronic stress, which has been associated with a heightened likelihood of more severe and persistent ADHD symptomatology (46), may exacerbate the condition’s core features that arise from atypical top-down influences of these brain regions on attentional and behavioral control (47, 48). ACEs constitute a significant risk factor for the emergence of severe ADHD symptomatology, with cumulative exposure exerting a particularly strong influence (49, 50). Conversely, children diagnosed with ADHD exhibit a disproportionately elevated risk of ACEs, indicating a bidirectional association between the two constructs when growing up (51). By demonstrating that ADHD symptoms in youth are associated with reduced parental involvement and more inconsistent disciplinary practices, these findings point to a plausible mechanism linking ADHD to an elevated risk of ACEs (52). ADHD-related behavioral characteristics may increase the likelihood of negative responses from caregivers and other adults, particularly in socioeconomically disadvantaged contexts (53, 54). Parenting a child with ADHD is often associated with heightened stress, which may contribute to increased family conflict and a greater risk of parental separation. In addition, children with ADHD may evoke more negative or inconsistent caregiving responses, thereby increasing their exposure to harsh parenting practices or relational adversity (54). These observations suggest that the increased exposure to ACEs among youth with ADHD may, at least in part, reflect reciprocal processes linking child behavior and environmental adversity (53). Such evocative gene-environment effects may give rise to a recursive, transgenerational cycle, in which adults with ADHD experience socioeconomic disadvantages, which in turn may exacerbate the expression of ADHD symptoms in their offspring (52, 53).

In recent years, an expanding body of research has concentrated on elucidating the complex interrelations between positive childhood experiences (PCEs) and both adaptive and maladaptive outcomes in adulthood (55). PCEs refer to nurturing relationships, safe and stable environments, opportunities for meaningful social engagement, and the development of emotional and cognitive skills that support resilience and well-being throughout life (56, 57) and have been associated with lower rates of depression, substance use, delinquent behavior, risky sexual behavior, as well as more favorable outcomes related to mental health, psychosocial functioning, and physical health. Notably, PCEs were consistently associated with lower levels of aggression (58).

Although cumulative PCEs in childhood may moderate the negative impact of ACEs (59), further meta-analytical results indicate that PCEs primarily exert direct, promotive effects on positive outcomes rather than significantly buffering adversity (60). Nevertheless, studies examining potential pathways from ACEs and PCEs to aggression remain limited. To the best of our knowledge, only one study has simultaneously examined the associations among ACEs, PCEs and aggression in adulthood (3). The findings reveal significant interactions between ACEs and PCEs in predicting anger among males and in predicting both aggression and anger among females. A recent cross-sectional study examining the relationship between ACEs, PCEs, and self-control in juvenile probationers documented that high ACE exposure consistently eroded self-control, whereas PCEs provided protective effects without fully mitigating the harm caused by elevated ACE levels (61). Furthermore, these findings indicate that PCEs may foster self-control independent of adversity, thereby underscoring the importance of minimizing ACEs (61). While each of these studies offers unique and valuable findings, both suffer from significant methodological limitations, including the use of inconsistent sources for ACE and PCE data, low reliability of measurement instruments (3, 61), artificial dichotomization of ACE/PCE scores (61), and gender imbalance in the samples (3).

Although evidence indicates that the influence of PCEs positively affects emotion regulation in adults with ADHD (62), research on long-term outcomes in adulthood remains limited. On the one hand, existing studies largely focus on adolescents with ADHD (63, 64), and on the other, PCEs and ACEs are often examined in isolation from one another (65–67). PCEs can partially buffer ACE effects (68); however, empirical evidence indicates that ACEs remain a very strong predictor of aggressive behavior and that PCE effects on aggression tend to be relatively small (3). Individuals with ADHD are disproportionately affected by ACEs (49, 50) and aggression is partially mediated not only by hyperactivity/impulsivity but also by inattention (69). Aggressive behavior in ADHD may arise from inattention and hyperactivity/impulsivity, whereby dysfunctional social information processing elicits feelings of threat or frustration and impulsivity facilitates aggressive responses (37). Inattention increases the risk of aggression through impaired interpretation of social cues and hostile attribution tendencies (70), whereas hyperactivity/impulsivity primarily promotes reactive, impulsive forms of aggression resulting from dysfunctional threat and stress processing (71, 72).

In individuals with ADHD, PCEs are therefore not expected to show an independent protective association with aggression beyond the effects of ACEs. To date, evidence is limited to findings that children with moderate to severe ADHD report fewer of the examined PCEs than those with mild ADHD (73), while no robust evidence indicates that PCEs are generally less prevalent in individuals with ADHD. We propose that PCEs promoting self-regulatory capacities (57, 74) may be particularly beneficial for individuals with ADHD.

Resilience theory encompasses several models that explain how promotive factors can counteract the adverse effects of risks, provide protection against them, or immunize young people from their impact (75). Resilience develops as a consequence of interactions among multiple ecosystems (76). Empirical findings indicate that a subset of children who have experienced ACEs nonetheless develop into healthy adults. This suggests the presence of effective protective factors during childhood development (77). Assuming an analytic framework guided by resilience theory, Zimmerman (78) based his approach on the compensatory model of resilience. Low aggression is operationalized in the present study as a resilience outcome, as it reflects positive adaptation in individuals who have been exposed to significant adversity (79, 80). In their meta-analysis on protective factors underlying resilience after childhood violence exposure, Yule et al. (80) emphasize that within resilience research, positive adaptation is conceptualized not only as the attainment of competencies or high levels of subjective well-being, but also as the absence or low presence of psychopathology. In a compensatory model, protective factors mitigate the effects of risk exposure by exerting their own beneficial influence on developmental outcomes. Within this framework, PCEs are conceptualized as protective factors that show a direct association with aggression that is conceptually and statistically distinct from the effects of ACEs. Importantly, this does not imply that PCEs operate in isolation from ACEs; rather, both types of experiences are assumed to act concurrently in shaping developmental outcomes. The application of the compensatory model (78) to our research would suggest that ACEs are likely to have a positive association with aggression, whereas PCEs should be associated with lower levels of aggression, thereby contributing to more favorable developmental outcomes even in the presence of ACEs.

Thus, the existing research leaves several important questions unanswered so far, especially regarding the association between ACEs, PCEs and aggression potential. These gaps are particularly consequential for vulnerable clinical populations, such as individuals with ADHD, for whom ACEs and PCEs may differentially shape behavioral regulation and aggression-related outcomes. A more sophisticated understanding of these associations could have therefore significant implications not only for research, but also for clinical and forensic assessments, as well as for policy, and practice. Given the limited empirical understanding of the complex relationships among these variables, the present study aims to investigate and compare their associations within ADHD and non-ADHD samples.

Based on the abovementioned theoretical and empirical foundations, we assumed that individuals with ADHD would report more ACEs than those without ADHD. Furthermore, individuals with ADHD were anticipated to display greater aggressive tendencies than the non-ADHD group. Higher ACE scores were expected to significantly predict adult aggression in both individuals with and without ADHD. We further hypothesized that among individuals without ADHD, PCEs would show a negative association with aggression once ACEs are controlled for, indicating a potential protective effect. In contrast, such a protective effect was not expected to emerge in adults with ADHD due to persistent impairments in self-regulatory functioning.

## 2 Materials and methods

### 2.1 Procedure

This study was embedded in a larger project examining clinically and forensically relevant risk factors [e.g., (69, 81)]. The mixed sample comprised individuals who had been assessed at the Institute for Forensic Psychology and Psychiatry, Saarland University, Homburg, in cooperation with the Section of Forensic Psychiatry and Psychotherapy at the University Medical Center of Johannes Gutenberg University Mainz, either for forensic evaluation or treatment or for ADHD assessment, as well as non-forensic, non-clinical participants recruited through direct personal contact. Participants completed a standardized set of self-report questionnaires in a paper-and-pencil format or through a digital platform using SoSci Survey (82), including the measurements considered for this study. Participation was voluntary and unpaid; informed consent was obtained. The study was conducted in accordance with the ethical standards of the Declaration of Helsinki and received approval from the ethics committee of the Medical Chamber of Saarland, Germany (Protocol Code: 58/22).

### 2.2 Sample

Between November 11, 2021, and May 5, 2025, a total of 415 individuals were assessed. Of these, 12 participants declined to have their data included in research analyses. Among the remaining 403 participants, 359 completed the questionnaires relevant to the present study. In this final sample, 170 individuals identified as female (47.4%), and 189 as male (52.6%). Participants’ ages ranged from 18 to 75 years (M = 35.31, SD = 13.25). The sample included 112 participants (31.2%) who had undergone forensic evaluation or therapy, 104 (29.0%) who had been assessed for possible ADHD, and 143 (39.9%) non-forensic/non-clinical participants.

### 2.3 Measures

#### 2.3.1 Attention-deficit/hyperactivity disorder

Current ADHD symptoms were assessed using the German self-report questionnaire for adult ADHD [ADHS-SB; (83)]. The ADHS-SB comprises 22 items, each rated on a four-point Likert scale (0 = does not apply, 1 = applies slightly/occasionally, 2 = applies moderately/often, 3 = applies strongly/almost always). The first nine items measure inattention, and the subsequent nine assess hyperactivity/impulsivity; together, these 18 items constitute the total score for current ADHD symptomatology. The remaining four items capture functional impairments and retrospective childhood symptoms.

The authors propose a cut-off value of 15 points on the total ADHD score to indicate clinically relevant ADHD, complementing the dimensional indices of symptom severity for inattention, hyperactivity/impulsivity, and overall symptomatology (83). Receiver operating characteristic analysis performed during scale development demonstrated that a cut-off score of 15 yielded sensitivity and specificity values of 77% and 75% (84), respectively, which the manual describes as a balanced compromise between these parameters (83). Moreover, a score of at least moderate severity on item 19 was required, indicating that the difficulties assessed in items 1 to 18 had already been present before the age of 12 years. These criteria were likewise applied in the present study to classify the sample with regard to the presence of ADHD. In the present (total) sample the questionnaire showed excellent internal consistency (Cronbach’s α = 0.952).

#### 2.3.2 Adverse childhood experiences

ACEs were assessed using the German 75-item version of the Maltreatment and Abuse Chronology of Exposure (MACE) scale (85, 86). Participants responded to a series of dichotomous yes-no questions, indicating whether they had been exposed to events across ten distinct ACE categories up to the age of 18: verbal parental abuse, non-verbal parental emotional abuse, parental physical abuse, emotional neglect, physical neglect, witnessing violence toward/between parents, witnessing violence toward siblings, emotional abuse by peers, physical abuse by peers, and sexual abuse. Previous research has demonstrated good psychometric properties for both the original and the German versions of the MACE scale (85, 86). However, due to the still insufficient empirical foundation, the subscale cut-off values proposed by Isele et al. (85) were not applied in the present study. Instead, a subscale was considered fulfilled if at least one item (experience) within a category was endorsed. This resulted in a total cumulative score ranging from 0 to 10, reflecting the total number of adversity categories encountered. Our approach aligns with more recent research, which predominantly conceptualizes childhood adversity using the cumulative risk framework. This framework aggregates the number of discrete ACEs an individual has encountered to produce a risk score, regardless of their specific type, duration, or severity, and employs this score as a predictor of developmental outcomes (87).

#### 2.3.3 Positive childhood experiences

PCEs up to the age of 18 were recorded based on the PCE framework by Baglivio and Wolff (68). We assessed a total of 11 binary-coded PCEs considering school environment, relationships with teachers, participation in school and extracurricular activities (such as community, cultural, or religious groups, clubs, sports), a sense of belonging to a community, the presence of a supportive network, a good relationship with at least one parent or a non-familial adult, and the involvement with non-delinquent friends only. We used a cumulative PCE sum score (0–11).

#### 2.3.4 Aggression

Aggression was measured using the Short Questionnaire for Assessing Factors of Aggression [K-FAF; Heubrock & Petermann (88)]. Items are rated on a five-point Likert scale (from 0 = not applicable to 5 = fully applicable). The scale measures self-reported impulsive aggression, reactive aggression, irritability, auto-aggression, and inhibition of aggression. The first three factors, as assessed for example by items such as “Sometimes I enjoy tormenting others.” “I’d rather strike than be a coward.” and “Unfortunately, I get angry quickly.” can be summed up into a comprehensive value, representing the aggregate of outwardly directed aggressiveness. As this was the focus of the present study, the remaining subscales auto-aggression and inhibition of aggression were not considered in the current analyses. In the present total sample, internal consistency for this sum score was high with Cronbach’s α = 0.924.

### 2.4 Statistical analyses

All statistical analyses were conducted using IBM SPSS Statistics for Windows, version 30. The general level of significance was set at p <.05. All tests were executed two-tailed. Internal consistency was deemed questionable with Cronbach’s α ≥ 0.60, acceptable with α ≥ 0.70, good with α ≥ 0.80, and excellent with α ≥ 0.90 (89). Group differences were examined using Shapiro–Wilk tests for normality, Mann–Whitney U tests for non-normally distributed variables, chi-square tests for categorical variables, and MANOVAs for dimensional variables. The effect size partial eta² (η_p_^2^) indicated a small effect with η_p_^2^ ≤ 0.01, a moderate effect with η_p_^2^ = 0.06, and a large effect with η_p_^2^ = 0.14 (90). To control for potential confounding effects, gender and age at the time of assessment were incorporated as covariates. Hierarchical linear regression analyses were conducted separately for individuals with and without ADHD. All explanatory variables were z-standardized.

## 3 Results

### 3.1 Descriptives

In the full sample, a total of 154 participants (42.9%) met the ADHS-SB cut-off for clinically relevant ADHD symptoms; accordingly, 205 participants (57.1%) were assigned to the non-ADHD group. The relatively high proportion of participants exceeding the ADHS-SB cut-off was expected, as part of the sample was recruited when individuals presented for an ADHD assessment. Furthermore, the prevalence of ADHD in forensic samples is typically considerably higher than in the general population.

**Table 1** summarizes the descriptive characteristics of the variables of interest for the total sample and separately by ADHD status. Observed scores covered a broad range across all study variables. The proportions of participants scoring at the minimum or maximum of the scales were low (all < 10%), and skewness (−0.46 to 0.86) and kurtosis (−1.02 to 0.67) values were within acceptable ranges, indicating no substantial floor or ceiling effects. Individuals with ADHD reported significantly higher levels of ACEs compared to those without ADHD, F (1, 353) = 42.99, *p* <.001, η^2p^ = .11. The ADHD group also reported higher aggression scores, F (1, 353) = 90.21, *p* <.001, η^2p^ = .20. In contrast, PCEs were significantly lower among adults with ADHD relative to those without ADHD, F (1, 353) = 4.73, *p* = .030, η^2p^ = .01.

**Table 1:** Descriptive distribution of the total values of the variables of interest.

	Total sample (N = 359)				ADHD (n = 154)				Non-ADHD (n = 205)				Group comparison		
Scores	M	SD	Range	Median	M	SD	Range	Median	M	SD	Range	Median	*F (1*,353)	*p*	η_p_^2^
ACEs	4.29	2.72	0.00 – 10.00	4.00	5.31	2.38	0.00 – 10.00	6.00	3.52	2.71	0.00 – 10.00	3.00	42.99	<.001	.11
PCEs	6.71	2.96	0.00 – 11.00	7.00	6.38	2.94	0.00 – 11.00	7.00	6.96	2.96	0.00 – 11.00	8.00	4.73	.030	.01
K-FAF	40.18	25.69	1.00 – 157.00	35.50	53.64	26.44	9.00 – 157.00	49.00	29.98	19.79	1.00 – 95.00	26.00	90.21	<.001	.20

M, Mean; SD, Standard deviation. Subsample sizes were reduced to n = 154 (ADHD) and n = 205 (Non-ADHD) in MANOVA. Self-reported aggression was assessed using the K-FAF.

Participants with ADHD (18–50 years; M = 32.64, SD = 10.92) were younger than those without ADHD (18–75 years; M = 37.32, SD = 14.53), as indicated by a Mann–Whitney U test (U = 13,336.50, Z = –2.52, p = .012). In the ADHD group, 77 individuals (50.0%) identified as female. A chi-square test of independence showed no significant association between gender and ADHD status, χ² (1) = 0.76, p = .384.

The reported types of ACEs were categorized according to the MACE subscales and stratified by ADHD status (see **Supplementary Table 1** in the **Supplementary**). Likewise, the reported PCE types were categorized by the presence of ADHD (see **Supplementary Table 2** in the **Supplementary**). **Supplementary Table 3** in the **Supplementary** presents the partial correlations between the variables of interest.

### 3.2 Hierarchical regression models

**Table 2** presents the hierarchical regression models examining the association between ACEs, PCEs, and aggressive behavior for adults with and without ADHD, controlling for age and gender. The assessment of multicollinearity revealed no issues; no tolerance value fell below.10, permitting the continuation of the regression analyses.

**Table 2:** Impact of adverse and positive childhood experiences on aggression in an ADHD and non-ADHD adult sample.

Group	Model	Δ*R²*	*p*	Predictors	*B*	95%*CI*	ß	*p*
Non-ADHD	Model A	.07	.002	ACEs	0.20	0.09, 0.30	0.25	.002
	Model B	.08	<.001	PCEs	-0.23	-0.33, -0.12	-0.30	<.001
	Model C	.11	<.001	ACEs	0.11	-0.01, 0.23	0.14	.072
				PCEs	-0.17	-0.29, -0.05	-0.22	.006
ADHD	Model A	.08	.008	ACEs	0.24	0.05, 0.43	0.20	.016
	Model B	.05	.020	PCEs	-0.16	-0.33, 0.00	-0.16	.053
	Model C	.09	.008	ACEs	0.20	0.00, 0.40	0.17	.049
				PCEs	-0.12	-0.29, 0.05	-0.11	.174

The analysis was controlled for age and gender. Female was coded as 0 and male was coded as 1.

#### 3.2.1 Non-ADHD group

In model A, ACEs significantly predicted higher aggression, ΔR² = .07, *p* = .002, with greater ACE exposure associated with elevated aggression levels (β = .25, *p* = .002). PCEs were associated with lower aggression (β = –.30, *p* <.001), increasing the explained variance to ΔR² = .08. The full model (model C) including both predictors showed that PCEs remained a significant negative predictor of aggression (β = –.22, *p* = .006), whereas the association between ACEs and aggression was attenuated and no longer statistically significant (β = .14, *p* = .072).

#### 3.2.2 ADHD group

Among adults with ADHD, ACEs were positively associated with aggression in model A, ΔR² = .08, *p* = .008 (β = .20, *p* = .016). In contrast to the non-ADHD group, PCEs did not significantly predict aggression in model B (β = –.16, *p* = .053), and this remained the case in the fully adjusted model c (β = –.11, *p* = .174). In the final model (model C), ACEs persisted as a significant predictor of aggression (β = .17, *p* = .049).

## 4 Discussion

The present study examined how ACEs and PCEs are associated with self-reported aggression in adulthood and whether these associations differ between individuals with and without ADHD. To our knowledge, despite its considerable relevance not only for research but also for clinical and societal implications, no previous study has simultaneously investigated ACEs, PCEs, and self-reported aggression as a function of ADHD status in adulthood. Thus, the present findings extend the limited empirical literature on the interplay between ACEs, PCEs, and self-reported aggression and address the lack of studies explicitly linking resilience-related constructs and ADHD (73).

Consistent with our expectations and previous research (3, 6, 91, 92), a positive association between cumulative ACEs and self-reported aggression was also observed regardless of ADHD status. This might be due to neurobiological alterations in brain regions and networks involved in stress regulation and emotional processing, including the amygdala, medial prefrontal cortex, hippocampus, and salience and frontoparietal networks (40, 41), which have been observed in individuals with and without ADHD, and that increase the risk of aggressive behavior (35).

In the present study, individuals with ADHD reported significantly higher ACE exposure and higher self-reported aggression scores than those without ADHD, supporting our first hypothesis. This pattern is consistent with previous research showing that people with ADHD more frequently report ACEs and exhibit elevated aggression and externalizing behavior (13, 14). ADHD is associated with difficulties in emotion regulation (32–34), which have been linked to aggressive behavior (35). Deficits in executive functions, particularly inhibitory control, may further contribute to impulsive and aggressive responses (36–38). ACEs have been associated with an increased likelihood of developing clinically relevant ADHD symptom severity, with cumulative ACE exposure exerting a particularly strong influence (49, 50, 93). Conversely, individuals with ADHD show higher rates of ACEs than those without the disorder, suggesting a bidirectional association between ADHD and childhood adversity (51). Recent studies indicate that ACEs are related to more severe ADHD symptoms and that these, in turn, are associated with higher self-reported aggression through reduced self-control (81). Furthermore, the hyperactivity/impulsivity dimension of ADHD has been identified as a mediator linking ACEs to adult aggression, particularly when cognitive reappraisal as an emotion-regulation strategy is used infrequently (69). Cognitive reappraisal involves altering the perceived meaning or self-relevance of a situation to modulate its emotional impact (94). This strategy has been linked to beneficial changes in affect, enhanced long-term well-being, and a reduced prevalence of psychopathological symptoms (95, 96).

In line with resilience theory, particularly the compensatory model (78), the present results show that among individuals without ADHD, higher cumulative PCE scores were associated with lower self-reported aggression, and the inclusion of PCEs attenuated the association between ACEs and self-reported aggression. This finding corresponds with studies showing that a higher number of protective factors is related to lower externalizing and violent behavior (97–99) and with research reporting negative associations between PCEs and aggression (58). It is also consistent with evidence that PCEs can partly attenuate adverse effects of ACEs (3, 59, 68), even if meta-analytic findings suggest that PCEs often show primarily direct promotive effects rather than strong buffering effects (60). Low aggression in the context of adversity can be conceptualized as a resilience outcome, reflecting positive adaptation despite exposure to risk (79, 80).

However, in line with our assumptions, PCEs did not show a statistically significant association with self-reported aggression in the ADHD group, neither when entered alone nor after controlling for ACEs, whereas ACEs remained a significant predictor in the fully adjusted regression. This suggests that cumulative ACEs were more strongly related to aggressive tendencies than cumulative PCEs among adults with ADHD. Previous research indicated that PCEs may vary by ADHD severity. Children with moderate-to-severe ADHD have been reported to experience fewer PCEs than those with milder symptoms (73), and children with high ACE exposure and low PCEs were more likely to show more severe ADHD symptoms (100). PCEs have generally been linked to resilience, self-control, and emotion regulation (101, 102) and may, in some contexts, promote adaptive outcomes among individuals with ADHD (62, 103). However, in the present study, PCEs did not demonstrate an independent protective association with self-reported aggression beyond ACEs in the ADHD subgroup, suggesting that beneficial effects of PCEs are observed only in the absence of ACEs among individuals with ADHD.

The present findings need to be considered in light of several strengths and limitations of our procedures. A central strength of the present study is the heterogeneous mixed-gender sample, which included participants from forensic, clinical, and non-forensic/non-clinical settings and covered a broad age range. This design increases variance in ACEs, PCEs, ADHD symptoms, and self-reported aggression and reduces the likelihood of floor or ceiling effects. Another strength is the simultaneous consideration of ACEs, PCEs, ADHD, and self-reported aggression within a single analytic framework. However, although our assessment was based on previous empirical foundations, there is no standardized definition or operationalization of either ACEs or PCEs (60, 104, 105). As our aggression questionnaire primarily focused on the readiness to engage in aggressive behavior rather than on overt aggressive acts (88), comparability with measures of direct criminal or violent conduct is limited. Additionally, our exclusive use of self-report to assess aggression may have influenced the results, given that offenders tend to underreport aggressive behaviors owing to social desirability biases and cognitive distortions. Accordingly, future research should include multi-informant assessments. Furthermore, it is important to consider that self-reported aggression, as assessed by the K-FAF, should primarily be interpreted as an aggressive behavioral tendency resulting from the interaction between dispositional factors and individual learning experiences. However, these dispositional propensities toward aggressive behaviors do not necessarily translate into actual aggressive actions. Nevertheless, the instrument has demonstrated criterion validity, as it reliably discriminates between healthy, non-delinquent individuals and offenders who have committed at least one violent offense and were placed either in penal custody or forensic psychiatric commitment by court judgment (88). However, this evidence does not extend to the prediction of individual aggressive incidents or clinical outcomes.

Moreover, all data were collected at only two research institutes, which restricts the generalizability of the findings, and the cross-sectional design does not allow causal inferences. All main variables (ACEs, PCEs, ADHD symptoms, and aggression) were assessed by self-report, which may be affected by recall bias, social desirability, and subjective perception. Adults with ADHD often recall their childhood symptoms incompletely and in a distorted manner, which may limit the validity of retrospective self-reports (106, 107). Moreover, recall accuracy appears to be related less to the severity of childhood symptoms than to the severity of current symptomatology (108). Of particular importance in this regard is the fact that the classification of participants into ADHD and non-ADHD groups was based on self-report ADHD questionnaire only, not on comprehensive clinical diagnoses. Although the ADHS-SB is a well-validated instrument with high internal consistency in the present sample, self-report alone does not provide a sufficient basis for clinical diagnosis (109) and should be seen as a supplement to clinical assessment. Furthermore, specific ADHD symptom profiles or clinical presentations were not analyzed separately, although differential pathways from inattention, hyperactivity/impulsivity, and poor self-control to offending have been suggested (110). Finally, possible comorbid psychiatric conditions could not be considered.

Despite these limitations, the present findings suggest several implications. The robust association between cumulative ACEs and self-reported aggression in both ADHD and non-ADHD adults underscores the importance of addressing ACEs as part of broader violence prevention and public health strategies. The observation that PCEs are associated with lower self-reported aggression in the non-ADHD group suggests that interventions may benefit from not only reducing ACEs but also systematically promoting PCEs. This is in line with recommendations to consider ACEs and PCEs cumulatively to inform targeted interventions that build on existing strengths and resources (111). Screening instruments for at-risk children and adolescents could be further developed to capture both adverse and positive experiences and to guide interventions that aim to reduce maladaptive experiences and foster protective activities that may be associated with lower self-reported aggression in adulthood.

With regard to ADHD, individuals affected may benefit from the implementation of screening procedures and evidence-based treatment for ADHD and trauma-related symptoms (including risk of aggressive behavior) to counteract maladaptive developmental trajectories (112). Furthermore, preventive efforts should focus on promoting PCEs among individuals without ADHD, whereas interventions for individuals with ADHD should prioritize the targeted reduction of ACEs.

In light of the findings presented in **Supplementary Table 1** and **S2** (**Supplementary Materials**), the implementation of evidence-based, family-focused interventions appears warranted to reduce offending behavior (113). However, persistent challenges, including limited long-term sustainability, motivational barriers, and high dropout rates, indicate that complementary systemic approaches may further enhance treatment effectiveness. Forensic Outpatient Systemic Therapy (FAST) represents one such approach, targeting aggressive and antisocial behavior in adolescents while strengthening family functioning (114, 115), thereby potentially mitigating ACEs and promoting PCEs through improved parent–child interactions and reduced family conflict. At the broader systemic level, FAST also aims to enhance social support (116) and reduce engagement with deviant peers (117). Accordingly, a comprehensive initial assessment should address psychosocial stressors affecting both the individual and the family system and include a structured evaluation of parent–child interactions and overall family relationships (118), ensuring that interventions are tailored to systemic needs. For individuals with a history of violent behavior, trauma-focused interventions may be indicated. Narrative exposure therapy (NET) offers a structured method to integrate traumatic experiences with aggression-related behavioral patterns and may contribute to sustained violence reduction and social reintegration (119).

In forensic settings, trauma-focused interventions, particularly phase 2 trauma-processing and individually administered programs, have been reported to reduce trauma-related symptoms (120), although evidence regarding their impact on later criminal behavior, especially among individuals with high ACE and low PCE exposure, remains limited (121). Pharmacological and psychotherapeutic treatment of ADHD may contribute to rehabilitation and a reduction in the likelihood of recidivism (122–124). In forensic assessment practice, it appears important to consider both ACEs and PCEs together with dynamic risk factors. The Risk-Need-Responsivity model emphasizes the identification and reduction of criminogenic needs (125), while the Good Lives Model (87) highlights the role of strengths and prosocial opportunities. The present findings are compatible with an integrated perspective that takes into account both risk reduction [e.g., ACEs, ADHD-related impairments] and the promotion of protective factors.

Future research should systematically account for socioeconomic status (SES) as a central contextual factor shaping the associations among ADHD, adverse and positive childhood experiences, and aggression. Low childhood SES is associated with increased risk for psychopathology, partly through greater exposure to adverse environmental conditions (126) and meta-analytic evidence links socioeconomic disadvantage to higher levels of antisocial behavior (127). Disadvantaged contexts are characterized by greater exposure to ACEs and reduced access to timely, appropriate mental health care (128), while children from low-income families remain underrepresented in psychosocial services and intervention research (129). In ADHD care, sustained clinical benefit is constrained by structural and attitudinal barriers that contribute to the underuse and premature discontinuation of multimodal treatment (130). Familial factors, including parental ADHD, depression, relationship instability, and parental adversity, may further hinder treatment engagement (131), although acute stress may initially promote help-seeking (132).

Yet, future research should also examine how specific patterns of ACEs and PCEs as well as their interactions relate to ADHD symptom dimensions and aggression over time. Longitudinal studies would allow a more precise analysis of temporal sequences and potential mediators, such as self-control and emotion regulation (61, 81, 110). In addition, further work is needed to explore other protective factors, such as access to mental health care, and to improve the conceptual and measurement consistency of ACEs and PCEs.

In summary, the present study indicates that ACEs are a robust correlate of self-reported aggression in adulthood in both ADHD and non-ADHD samples, whereas PCEs show a compensatory association with lower self-reported aggression only in individuals without ADHD. These findings contribute to a more nuanced understanding of the associations between ACEs, PCEs, ADHD, and self-reported aggression and may inform prevention and intervention approaches that consider both adverse and positive childhood experiences.

## Data Availability

The data analyzed in this study are subject to the following licenses/restrictions: The datasets presented in this article are not readily available because of the specific confidentiality of the assessed clinical and forensic information. Scientists wishing to use them for non-commercial purposes are kindly asked to contact the present authors to frame individual agreements. Requests to access these datasets should be directed to JM, johannes.merscher@uni-saarland.de.
