# Supplementary material for: Risk and resilience: adverse and positive childhood experiences and aggression in adults with and without ADHD
Source: Front Psychiatry. 2026 Mar 10;17:1759667. doi: 10.3389/fpsyt.2026.1759667 (PMC13008880; doi:10.3389/fpsyt.2026.1759667)
Supplement: Supplementary file 1 [file SupplementaryFile1.docx]

**Table S1** Reported Types of Adverse Childhood Experiences

|  | ADHD (n) | ADHD (%) | Non-ADHD (n) | Non-ADHD (%) |  |
| --- | --- | --- | --- | --- | --- |
| Parental physical abuse | 90 | 58.4 | 89 | 43.4 |  |
| Parental verbal abuse | 119 | 77.3 | 100 | 48.8 |  |
| Parental nonv. emotional abuse | 114 | 74.0 | 96 | 46.8 |  |
| Sexual abuse | 42 | 27.3 | 28 | 13.7 |  |
| Emotional neglect | 136 | 88.3 | 136 | 66.3 |  |
| Physical neglect | 78 | 50.6 | 82 | 40.0 |  |
| Witnessed physical violence toward parents | 23 | 14.9 | 33 | 16.1 |  |
| Witnessed violence toward siblings | 36 | 23.4 | 33 | 16.1 |  |
| Peer emotional violence | 121 | 78.6 | 98 | 47.8 |  |
| Peer physical violence | 59 | 38.3 | 26 | 12.7 |  |
| *Note.* *Proportion of reported types of ACEs, presented separately for the ADHD (n=154) and non-ADHD (n=205) samples.* | | | | | |

**Table S2** Reported Types of Positive Childhood Experiences

|  | ADHD (n) | ADHD (%) | Non-ADHD (n) | Non-ADHD (%) |  |
| --- | --- | --- | --- | --- | --- |
| Finds school encouraging | 76 | 49.4 | 142 | 69.3 |  |
| Prosocial School Bonds | 70 | 45.5 | 101 | 49.3 |  |
| Involved in School Activities | 116 | 75.3 | 152 | 74.1 |  |
| Involved in structured activities | 115 | 74.7 | 159 | 77.6 |  |
| Positive adult relationships | 61 | 39.6 | 101 | 49.3 |  |
| Prosocial friends | 90 | 58.4 | 112 | 54.6 |  |
| Prosocial community ties | 69 | 44.8 | 97 | 47.3 |  |
| Social support for family | 76 | 49.4 | 105 | 51.2 |  |
| Supportive family | 105 | 68.2 | 161 | 78.5 |  |
| Family involvement | 91 | 59.1 | 137 | 66.8 |  |
| Close parental relationship | 113 | 73.4 | 159 | 77.6 |  |
| *Note.* *Proportion of reported types of PCEs, presented separately for the ADHD (n=154) and non-ADHD (n=205) samples.* | | | | | |

|  | (1) | (2) | (3) |
| --- | --- | --- | --- |
| ACEs (1) | - | -.428***  [-.512, -.331] | .349***  [.257, .430] |
| PCEs (2) |  | - | -.257***  [-.358, -.158] |
| K-FAF (3) |  |  | - |

Table **S3** Partial correlations between the variables of interest (bias-corrected and accelerated) with 95% confidence intervals for the total sample

*Note.* ****p < .001. Analyses based on z-standardized values with age and gender as covariates. N = 359. Self-reported aggression was assessed using the K-FAF.*
